# Electron specific absorbed fractions for the ICRP family of mesh-type reference computational phantoms

**DOI:** 10.1088/1361-6560/aeb799

**Published:** 2026-11-09

**Authors:** Wyatt W Smither, Chansoo Choi, Bangho Shin, Derek W Jokisch, Emmanuel M Mate-Kole, Shaheen A Dewji, Wesley E Bolch

**Affiliations:** 1 Department of Biomedical Engineering, University of Florida, Gainesville, FL, United States of America; 2 Department of Nuclear Engineering, Jeju National University, Jeju, Republic of Korea; 3 Department of Physics and Engineering, Francis Marion University, Florence, SC, United States of America; 4 The Georgia Institute of Technology, George W. Woodruff School of Mechanical Eng., Atlanta, GA, United States of America; 5 Abilene Christian University, Abilene, TX, United States of America

**Keywords:** ICRP reference computational phantoms, electron specific absorbed fractions, internal dosimetry

## Abstract

*Objective.* To provide electron specific absorbed fractions (SAFs) using the mesh-type reference computational phantoms (MRCPs) developed by the International Commission on Radiological Protection (ICRP). *Approach.* The ICRP family of MRCPs from Publications 145 and 156 supersedes the voxel-type reference computational phantoms (VRCPs) used in ICRP Publications 133 and 155. Unlike the VRCPs, the MRCPs can explicitly define micrometer-scale radiosensitive target tissues and include intra-organ blood content. Electron SAFs were computed for all 12 MRCPs using the Particle and Heavy Ion Transport code System (PHITS) Monte Carlo code, covering 86 source regions and 62 target regions. Total electron SAFs were obtained using a component-based method that separates collisional and radiative contributions, with the latter derived from our companion photon SAF datasets. Results for the adult male MRCP were independently verified against Geant4 calculations. *Main Results.* The MRCP-based electron SAF datasets showed agreement with ICRP Publications 133 and 155 for self-absorption geometries across the full energy range. For crossfire geometries, differences were observed and are likely attributable to several factors, including revised organ geometries, the representation of micrometer-scale target tissue layers, the use of different photon datasets for the radiative SAFs, and differing low-energy extrapolation methods. Age-dependent trends were observed, with younger reference individuals often yielding higher SAF values. General agreement was observed between PHITS and Geant4 across the full energy range and source-target combinations considered. *Significance.* To the authors’ knowledge, this is the first study to provide complete electron SAF datasets spanning all ICRP MRCPs with expanded source-target combinations.

## 1. Introduction

Internal radiation exposure arises when radionuclides enter the body through pathways such as medical administration, inhalation, ingestion, or wound contamination (ICRP 1991, 2007). Once incorporated, radionuclides are distributed and retained in specific organs and tissues, as described by the International Commission on Radiological Protection (ICRP) systemic, alimentary tract, and respiratory tract biokinetic models (ICRP 1994, 2006, 2015, 2016b, 2017, 2019, 2022, 2024b). Accurate estimation of internal radiation doses is central to radiation protection and supports the assessment of effective and absorbed doses in occupational and medical contexts. The mean absorbed dose delivered to a target tissue over a dose-integration period can be expressed as:

\begin{equation*}D\left( {{r_{\mathrm{T}}},{T_{\mathrm{D}}}} \right) = \mathop {\mathop \sum \nolimits }\limits_{{r_{\mathrm{S}}}} \mathop \int \limits_0^{{T_{\mathrm{D}}}} A\left( {{r_{\mathrm{S}}},t} \right)S\left( {{r_{\mathrm{T}}} \leftarrow {r_{\mathrm{S}}},t} \right){\mathrm{d}}t\end{equation*}

where $D\left( {{r_{\mathrm{T}}},{T_{\mathrm{D}}}} \right)$ is the mean absorbed dose to target tissue ${r_{\mathrm{T}}}$ resulting from radioactive material uniformly distributed in source tissue ${r_{\mathrm{S}}}$ over the dose-integration period ${T_{\mathrm{D}}}$, $A\left( {{r_{\mathrm{S}}},t} \right)$ is the time-dependent activity of the radionuclide in ${r_{\mathrm{S}}}$, and $S\left( {{r_{\mathrm{T}}} \leftarrow {r_{\mathrm{S}}},t} \right)$ is the radionuclide-specific absorbed dose to ${r_{\mathrm{T}}}$ per nuclear transformation in ${r_{\mathrm{S}}}$ at time t. The *S* value (or *S* coefficient) accounts for both the radiation emission characteristics of the radionuclide and the anatomical geometry of the source-target pair. Consistent with the ICRP formalism, it is treated as time-independent, $S\left( {{r_{\mathrm{T}}} \leftarrow {r_{\mathrm{S}}}} \right)$, for adults and as time-dependent, $S\left( {{r_{\mathrm{T}}} \leftarrow {r_{\mathrm{S}}},t} \right)$, for younger individuals over ${T_{\mathrm{D}}}$. The *S* value can be computed by:

\begin{equation*}S\left( {{r_{\mathrm{T}}} \leftarrow {r_{\mathrm{S}}},t} \right) = \mathop {\mathop \sum \nolimits }\limits_i {E_i}{Y_i}{{\Phi }}\left( {{r_{\mathrm{T}}} \leftarrow {r_{\mathrm{S}}},{E_i}} \right)\end{equation*}

where the summation runs over all nuclear transitions *i* of the radionuclide, ${E_i}$ is the energy of transition *i*, ${Y_i}$ is the yield of that transition per nuclear transformation, and ${{\Phi }}\left( {{r_{\mathrm{T}}} \leftarrow {r_{\mathrm{S}}},{E_i}} \right)$ is the specific absorbed fraction (SAF) for target tissue ${r_{\mathrm{T}}}$ from source tissue ${r_{\mathrm{S}}}$ at energy ${E_i}$. Further, the SAF is given by:

\begin{equation*}{{\Phi }}\left( {{r_{\mathrm{T}}} \leftarrow {r_{\mathrm{S}}},{E_i}} \right) = \frac{{\phi \left( {{r_{\mathrm{T}}} \leftarrow {r_{\mathrm{S}}},{E_i}} \right)}}{{m\left( {{r_{\mathrm{T}}}} \right)}}\end{equation*}

where $\phi \left( {{r_{\mathrm{T}}} \leftarrow {r_{\mathrm{S}}},{E_i}} \right)$ is the absorbed fraction (AF), defined as the fraction of the energy ${E_i}$ emitted within source tissue ${r_{\mathrm{S}}}$ that is deposited in target tissue ${r_{\mathrm{T}}}$, and $m\left( {{r_{\mathrm{T}}}} \right)$ is the mass of target tissue ${r_{\mathrm{T}}}$.

In early estimations of internal doses from electrons, simplifying assumptions were made. The foundational framework for internal dosimetry was established by the MIRD Committee in Pamphlet No. 1 (Loevinger and Berman 1968), which introduced the schema for absorbed dose calculations from biologically distributed radionuclides. Snyder *et al* (1975) used a simple characterization of the electron SAF, in which values were set to the reciprocal of the target mass (corresponding to an AF equal to unity) when the source and target were identical, and to zero when the source and target were well separated across the entire energy range. For hollow organs in which the contents served as the source region, the SAF across the energy range was found by setting the AF to one-half and dividing by the mass of the source contents. For intraskeletal irradiation to the red (active) bone marrow, the dosimetry methods by Spiers (1968, 1969, 1974) and Spiers and Vaughan (1976) were followed. These methods assumed that the cortical and trabecular bones are each uniformly labeled by a beta-emitting radionuclide, and AFs were then estimated using formulae based on ratios of electron stopping power in various media.

These simplifying assumptions were formally codified in ICRP Publication 30 (ICRP 1979), which adopted the convention that all electron and beta-particle energy was deposited locally within the source organ, except for intraskeletal dosimetry cases. This approach was further perpetuated in the age-dependent stylized series of Cristy and Eckerman (1987), which extended the ORNL phantom framework to six reference ages and provided photon SAF data across this age range. No explicit electron SAFs were computed for any of these phantoms, and the local energy deposition assumption remained the standard for electron dosimetry.

The transition toward explicit electron radiation transport came along with the advent of voxel-based computational phantoms derived from tomographic medical imaging data, which provided anatomically realistic organ geometries and allowed for the revisitation of the earlier analytical approximations. As radiation transport codes could now handle charged particles and became more widely accessible, it was increasingly feasible to abandon the local deposition assumption and directly simulate electron transport in a manner analogous to that long established for photons. Radiation transport for charged particles was subsequently applied to human voxel computational phantoms, enabling the explicit calculation of electron SAFs for the first time (Chao and Xu 2001, Han *et al* 2006, Hadid *et al* 2010, Zankl *et al* 2012). These studies demonstrated that dose estimates for electron-emitting radionuclides differed meaningfully from those derived under the historical assumptions, which accounted only for organ self-dose and neglected cross-organ energy deposition. In particular, it was shown that the local absorption assumption broke down at electron energies above approximately 300–500 keV, where high-energy electrons were capable of traversing organ boundaries and depositing a non-negligible fraction of their energy in adjacent tissues.

Comprehensive SAF datasets for internally emitted photons, electrons, alpha particles, and fission-spectrum neutrons have recently been computed and compiled by the ICRP in Publications 133 and 155, the latter extending coverage to pediatric reference individuals from newborn to 15 years of age (ICRP 2016a, 2023). These datasets were generated primarily using voxel-type reference computational phantoms (VRCPs) derived from CT image datasets of real individuals, with organ geometries adjusted to conform to ICRP reference masses (ICRP 2002, 2009, 2020a). While the VRCPs represented a substantial advancement at the time of their development, several anatomical and geometric limitations have since been identified. Organ volumes were defined to accommodate parenchymal tissue only, omitting intra-organ blood content, requiring post-hoc corrections to VRCP-derived SAF values for self-absorption cases. Voxel resolution precluded the direct modeling of micrometer-scale radiosensitive target layers, such as those lining the respiratory and alimentary tracts, the basal layer of the skin, and the urinary bladder wall, necessitating their treatment through separate stylized geometric models external to the whole-body phantoms or reliance on surrogate targets. Additional inconsistencies in the skeletal system, colon, and small intestine geometry have also been documented (ICRP 2016a, 2023). These shortcomings were subsequently addressed through the ICRP’s development and publication of the mesh-type reference computational phantoms (MRCPs) for adult and pediatric reference individuals in ICRP Publications 145 and 156, respectively (ICRP 2020b, 2024a).

Unlike higher energy photons, electrons are short-range charged particles whose energy deposition is more localized, making computed SAF values acutely sensitive to the accuracy of source and target regions. The MRCPs directly address this by providing smooth, anatomically realistic organ boundaries in high-fidelity tetrahedral mesh format and the explicit incorporation of thin radiosensitive target layers within the MRCPs. Building upon our recent study which provided comprehensive photon SAF datasets using the ICRP MRCPs (Smither *et al* 2026b), the present study extends this work to generate, to the authors’ knowledge, the first complete dataset of reference electron SAFs derived from the full ICRP family of adult and pediatric MRCPs.

## 2. Materials and methods

### 2.1. ICRP MRCPs

The ICRP MRCPs were used as the anatomical basis for all electron SAF calculations in this work with the exception of intraskeletal geometries (see section 2.3). Briefly, the MRCPs were developed by converting the ICRP VRCPs into tetrahedral mesh format, incorporating anatomically realistic models of micrometer-scale radiosensitive target tissues, and correcting several known anatomical deficiencies of the VRCPs, including the explicit addition of intra-organ blood content and revised models of the skeletal system, colon, small intestine, extrathoracic airways, eyes, teeth, and thyroid (ICRP 2020b, 2024a, Choi *et al* 2021, 2022, 2023, Han *et al* 2021, Shin *et al* 2021, Yeom *et al* 2022). The ICRP family of MRCPs are shown in figure 1.

**Figure 1.**
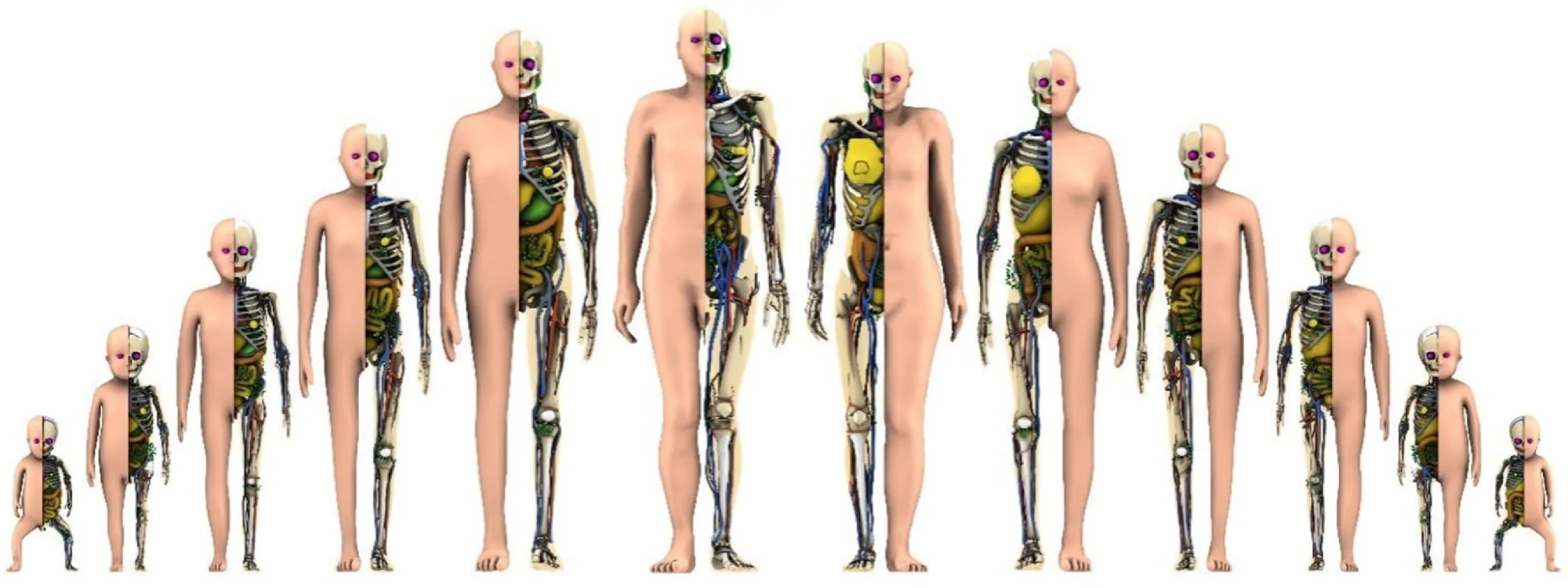
ICRP mesh-type reference computational phantoms (MRCPs) for male (left) and female (right) individuals at newborn, 1, 5, 10, and 15 years of age, and adult.

From the perspective of internal electron dosimetry specifically, the tetrahedral mesh format offers advantages beyond those relevant to photon transport. The smooth organ boundaries afforded by mesh geometries reduce artifacts in dose calculations for short-range particles, where greater proportions of energy deposition are mainly concentrated near target interfaces. The explicit definition of thin radiosensitive target layers within the MRCPs as opposed to using surrogate tissues or external stylized models enables self-consistent electron SAF calculations. Additionally, the incorporation of intra-organ blood in the MRCPs caused an increase in the volume of organs, altering the conformational relationship between organ boundaries.

### 2.2. Electron SAFs from non-skeletal source regions

Electron SAFs were computed for all defined source and target regions listed in tables 1 and 2, respectively, using the ICRP adult and pediatric MRCPs described in ICRP Publications 145 and 156, for reference male and female individuals at newborn, 1, 5, 10, 15 years of age, and adult. As with the photon work, anatomically realistic source and target definitions were employed throughout, including the explicit treatment of micrometer-scale radiosensitive target tissues and expanded source-target region combinations not previously available in the VRCP-based datasets.

**Table 1.** List of source regions and their acronyms.

Source region	Acronym	Source region	Acronym
Oral cavity	O-cavity	Major blood vessels	MBV
Oral mucosa	O-mucosa	Blood in heart	Ht-cont
Teeth surface	Teeth-S	Total blood	Blood
Teeth volume	Teeth-V	Cortical bone surface	C-bone-S
Tongue	Tongue	Cortical bone volume	C-bone-V
Tonsils	Tonsils	Trabecular bone surface	T-bone-S
Oesophagus fast	Oesophag-f	Trabecular bone volume	T-bone-V
Oesophagus slow	Oesophag-s	Cortical bone marrow	C-marrow
Oesophagus	Oesophag-w	Red (active) bone marrow	R-marrow
Stomach contents	St-cont	Trabecular bone marrow	T-marrow
Stomach wall	St-wall	Yellow (inactive) bone marrow	Y-marrow
Stomach mucosa	St-mucosa	Brain	Brain
Small intestine contents	SI-cont	Breast adipose + breast glandular	Breast
Small intestine villi	SI-villi	Lens of eye	Eye-lens
Small intestine wall	SI-wall	Gallbladder	GB-wall
Small intestine mucosa	SI-mucosa	Gallbladder contents	GB-cont
Right colon contents	RC-cont	Heart	Ht-wall
Right colon wall	RC-wall	Right kidney cortex	RKidney-C
Right colon mucosa	RC-mucosa	Right kidney medulla	RKidney-M
Left colon contents	LC-cont	Right kidney pelvis	RKidney-P
Left colon wall	LC-wall	Left kidney cortex	LKidney-C
Left colon mucosa	LC-mucosa	Left kidney medulla	LKidney-M
Rectosigmoid colon contents	RS-cont	Left kidney pelvis	LKidney-P
Rectosigmoid colon wall	RS-wall	Right kidney + left kidney	Kidneys
Rectosigmoid colon mucosa	RS-mucosa	Liver	Liver
Anterior nasal passage surface	ET_1_-sur	Systemic lymph nodes	LN-Sys
Posterior nasal passage surface	ET_2_-sur	Muscle	Muscle
Anterior nasal passage wall	ET_1_-wall	Right ovary + left ovary	Ovaries
Posterior nasal passage wall	ET_2_-wall	Pancreas	Pancreas
Posterior nasal passage bound region	ET_2_-bnd	Pituitary gland	P-gland
Posterior nasal passage sequestered region	ET_2_-seq	Prostate	Prostate
Extrathoracic lymph nodes	LN-ET	Salivary glands	S-glands
Bronchi	Bronchi	Skin	Skin
Bronchi bound region	Bronchi-b	Spleen	Spleen
Bronchi sequestered region	Bronchi-q	Testes	Testes
Bronchiole	Brchiole	Thymus	Thymus
Bronchiolar bound region	Brchiole-b	Thyroid	Thyroid
Bronchiolar sequestered region	Brchiole-q	Ureters	Ureters
Alveolar interstitium	ALV	Urinary bladder	UB-wall
Lung tissue	Lung-Tis	Urinary bladder content	UB-cont
Respiratory tract air	RT-Air	Uterus/cervix	Uterus
Thoracic lymph nodes	LN-Th	Adipose/residual tissue	Adipose
Right adrenal + left adrenal	Adrenals	Cartilage	Cartilage

**Table 2.** List of target regions and their acronyms.

Target region	Acronym	Target region	Acronym
Oral mucosa	O-mucosa	Thyroid	Thyroid
Oesophagus wall	Oesophagus	Breast adipose + breast glandular	Breast
Oesophagus basal cells	Oesophag-bas	Thymus	Thymus
Stomach wall	St-wall	Heart wall	Ht-wall
Stomach stem cells	St-stem	Right adrenal + left adrenal	Adrenals
Small intestine wall	SI-wall	Liver	Liver
Small intestine stem cells	SI-stem	Pancreas	Pancreas
Colon wall	Colon-wall	Right kidney + left kidney	Kidneys
Colon stem cells	Colon-stem	Right kidney cortex	RKidney-C
Right colon wall (ascending + right transverse)	RC-wall	Right kidney medulla	RKidney-M
Right colon stem cells (ascending + right transverse)	RC-stem	Right kidney pelvis	RKidney-P
Left colon wall (left transverse + descending)	LC-wall	Left kidney cortex	LKidney-C
Left colon stem cells (left transverse + descending)	LC-stem	Left kidney medulla	LKidney-M
Rectosigmoid colon wall (sigmoid + rectum)	RS-wall	Left kidney pelvis	LKidney-P
Rectosigmoid colon stem cells (sigmoid + rectum)	RS-stem	Spleen	Spleen
Anterior nasal passage wall	ET_1_-wall	Gallbladder wall	GB-wall
Posterior nasal passage wall	ET_2_-wall	Ureters	Ureters
Anterior nasal passage basal cells	ET_1_-bas	Urinary bladder wall	UB-wall
Posterior nasal passage basal cells	ET_2_-bas	Urinary bladder basal cells	UB-bas
Extrathoracic lymph nodes	LN-ET	Systemic lymph nodes	LN-Sys
Bronchi basal cells	Bronch-bas (Gen 1)	Skin	Skin
Bronchi sequestered region	Bronch-sec (Gen 1)	Skin basal cells	Skin-bas
Bronchiolar secretory cells	Bchiol-sec	Adipose/residual tissue	Adipose
Alveolar interstitium	AI	Muscle	Muscle
Thoracic lymph nodes	LN-Th	Spinal cord	Sp-cord
Brain	Brain	Red (active) bone marrow	R-marrow
Lens of eye	Eye-lens	50-μm endosteal region	Endost-BS
Pituitary gland	P-gland	Right ovary + left ovary	Ovaries
Tongue	Tongue	Uterus/cervix	Uterus
Tonsils	Tonsils	Prostate	Prostate
Salivary glands	S-glands	Testes	Testes

Schwarz *et al* (2021a) and ICRP Publication 155 (ICRP 2023) provide an expression for the total electron SAF, which is computed by:

\begin{equation*}{{{\Phi }}_{{\mathrm{tot}}}}\left( {{r_{\mathrm{T}}} \leftarrow {r_{\mathrm{S}}},{E_i}} \right) = {{{\Phi }}_{{\mathrm{col}}}}\left( {{r_{\mathrm{T}}} \leftarrow {r_{\mathrm{S}}},{E_i}} \right) + {{{\Phi }}_{{\mathrm{rad}}}}\left( {{r_{\mathrm{T}}} \leftarrow {r_{\mathrm{S}}},{E_i}} \right)\end{equation*}

where ${{{\Phi }}_{{\mathrm{col}}}}\left( {{r_{\mathrm{T}}} \leftarrow {r_{\mathrm{S}}},{E_i}} \right)$ is the component of the electron SAF due to collisional interactions and ${{{\Phi }}_{{\mathrm{rad}}}}\left( {{r_{\mathrm{T}}} \leftarrow {r_{\mathrm{S}}},{E_i}} \right)$ is the component of the electron SAF due to bremsstrahlung x-rays produced in source tissues.

The collisional component of the electron SAF for non-skeletal sources and targets is computed by equation (3). ICRP Publication 116 (ICRP 2010) provides a framework for computing the collisional component of the electron SAF for red bone marrow (R-marrow) and endosteal surface (Endost-BS) targets from source regions originating outside of the skeletal tissues:

\begin{equation*}{{{\Phi }}_{{\mathrm{col}}}}\left( {{\mathrm{R-marrow}} \leftarrow {r_{\mathrm{S}}},{E_i}} \right) = \mathop {\mathop \sum \nolimits }\limits_x \frac{{m\left( {{\mathrm{R-marrow}},x} \right)}}{{m\left( {{\mathrm{R-marrow}}} \right)}}{{{\Phi }}_{{\mathrm{col}}}}\left( {{\mathrm{SP}},x} \right) + \mathop {\mathop \sum \nolimits }\limits_x \frac{{m\left( {{\mathrm{R-marrow}},x} \right)}}{{m\left( {{\mathrm{R-marrow}}} \right)}}{{{\Phi }}_{{\mathrm{col}}}}\left( {{\mathrm{MM}},x} \right)\end{equation*}

\begin{equation*}{{{\Phi }}_{{\mathrm{col}}}}\left( {{\mathrm{Endost-BS}} \leftarrow {r_{\mathrm{S}}},{E_i}} \right) = \mathop {\mathop \sum \nolimits }\limits_x \frac{{m\left( {{\mathrm{Endost-BS}},x} \right)}}{{m\left( {{\mathrm{Endost-BS}}} \right)}}{{{\Phi }}_{{\mathrm{col}}}}\left( {{\mathrm{SP}},x} \right) + \mathop {\mathop \sum \nolimits }\limits_x \frac{{m\left( {{\mathrm{Endost-BS}},x} \right)}}{{m\left( {{\mathrm{Endost-BS}}} \right)}}{{{\Phi }}_{{\mathrm{col}}}}\left( {{\mathrm{MM}},x} \right)\end{equation*}

where $m(\mathrm{R-marrow},x)$ and $m(\mathrm{Endost-BS},x)$ are the mass of the red bone marrow and endosteal surface in skeletal site x, respectively, $m(\mathrm{R-marrow})$ and $m(\mathrm{Endost-BS})$ are the total mass of red bone marrow and endosteal surface across all skeletal sites, respectively, and ${\Phi_{\mathrm{col}}}(\mathrm{SP} ,x)$ and ${\Phi_{\mathrm{col}}}(\mathrm{MM} ,x)$ are the collisional SAF to the spongiosa and medullary marrow at bone site x, respectively.

The radiative component of the electron SAF is computed as the bremsstrahlung-spectrum-weighted photon SAF, where each bin’s contribution is weighted by the number of photons produced per source electron and their energy, normalized to the initial electron energy as previously described by Wayson *et al* (2012) and later adopted by Schwarz *et al* (2021a) and the ICRP (2023). The method of independently computing the collisional and radiative components of the electron SAF is demonstrated by Wayson *et al* (2012) to improve statistical reliability of the low-yield bremsstrahlung component to deposited energy in faraway target tissues, and was utilized in this study. This method necessarily assumes that only bremsstrahlung photons produced in the source region contribute to the radiative SAF and neglects any bremsstrahlung photons produced outside the source region. Studies by Wayson *et al* (2012) and this work (see section 3.2) demonstrate that total electron SAF results from a single Monte Carlo simulation where photon transport is employed and bremsstrahlung photons are able to be produced outside of the source region are comparable to those obtained by the component-based method. In discrete form, the radiative electron SAF is computed by

\begin{equation*}{{{\Phi }}_{{\mathrm{rad}}}}\left( {{r_{\mathrm{T}}} \leftarrow {r_{\mathrm{S}}},{E_i}} \right) = \frac{1}{{{E_i}}}\sum\limits_n {B_n}\left( {{E_i}} \right) \cdot {E_{P,n}} \cdot {{{\Phi }}_P}\left( {{r_{\mathrm{T}}} \leftarrow {r_{\mathrm{S}}},{E_{P,n}}} \right)\end{equation*}

where ${B_n}({E_i})$ is the number of bremsstrahlung photons produced in the nth photon energy bin per source electron of energy ${E_i}$, ${E_{P,n}}$ is the representative energy of the nth bin, taken here as the bin midpoint, and ${{{\Phi }}_P}\left( {{r_{\mathrm{T}}} \leftarrow {r_{\mathrm{S}}},{E_{P,n}}} \right)$ is the photon SAF evaluated at ${E_{P,n}}$, obtained by piecewise cubic Hermite interpolating polynomial (PCHIP) interpolation from our recent study on photon SAFs.

Shin *et al* (2025) recently published updated SAFs and *S* values for intra-human respiratory tract model (HRTM) geometries using the ICRP adult and pediatric MRCPs, incorporating highly detailed respiratory tract structures in both tetrahedral mesh and constructive solid geometry (CSG) formats. The resulting SAFs and *S* values demonstrated pronounced age dependence and were generally larger than those reported in ICRP Publications 133 and 155, attributed to differences in target masses and airway branching geometry. The electron SAF dataset from Shin *et al* (2025) was adopted for use in the present study and appended to the SAF datasets reported here without modification. The adopted values comprise all four extrathoracic source regions (ET1-sur, ET2-sur, ET2-bnd, ET2-seq) paired with both extrathoracic targets (ET1-bas, ET2-bas), and all seven thoracic source regions (Bronchi, Bronchi-b, Bronchi-q, Brchiole, Brchiole-b, Brchiole-q, ALV) paired with all four thoracic targets (Bronch-bas, Bronch-sec, Bchiol-sec, AI), giving 36 source-target combinations in total. As the Shin *et al* (2025) dataset did not include a lung tissue (Lung-Tis) source region, additional Geant4 Monte Carlo simulations for bronchial wall and bronchiolar wall sources were conducted accordingly in the present study.

To assess crossfire doses from HRTM source regions to non-HRTM targets, phase space files were generated from the CSG-format airway models and integrated into the homogenized lung models of the MRCPs, as previously described in our recent photon study. Discrete phase space files were produced for the alveolar interstitium, respiratory tract air, and each bronchial and bronchiolar region, and subsequently imported into the radiation transport simulation for electron SAF calculations. Bremsstrahlung spectra for these sources were taken from those photons which were produced in the MRCP-based homogenized lung regions.

### 2.3. Electron SAFs from skeletal source regions

ICRP Publication 155 outlines the methodology used to compute intraskeletal electron SAFs for pediatric and adult reference individuals, derived from image-based microstructure models that could not be captured in the VRCPs and are likewise not captured in the MRCPs (Pafundi *et al* 2010, Hough *et al* 2011, O’Reilly *et al* 2016). For the newborn reference model, electron SAFs were derived from Pafundi (2009) and were based on paired-image radiation transport simulations combining CT and *μ*CT imaging of spongiosa cores from five skeletal sites in two female newborn cadavers (Pafundi *et al* 2009, 2010). Where voxelized models were unavailable for certain skeletal sites, SAF values were estimated using weighting schemes originally proposed by Whitwell (1973). The 15-year-old model relied on analogous simulations across 26 skeletal sites from an 18-year-old male cadaver, following methods established from the University of Florida’s reference adult male and adult female phantoms (Hough *et al* 2011, O’Reilly *et al* 2016).

For the 1-, 5-, and 10-year-old reference models, physical spongiosa samples were unavailable, necessitating a two-stage approach: macroscale electron transport was first conducted within the reference VRCPs to characterize source regions in cortical bone, trabecular spongiosa, and medullary cavities, followed by microscale transport using optical pathlength distributions reported by Beddoe *et al* (1976) for children of approximately comparable ages, implemented in simulated 3D geometries as described by Watchman *et al* (2005). The 5-year-old model used age-interpolated pathlength distributions between the available datasets. SAFs across all pediatric ages were presented as a function of age without sex differentiation.

The present study utilizes the intraskeletal electron SAFs reported in ICRP Publications 133 and 155 (ICRP 2016a, 2023). These comprise all bone source regions (C-bone-V, T-bone-V, C-bone-S, T-bone-S, C-marrow, R-marrow, T-marrow, Y-marrow) paired with both skeletal targets (Endost-BS, R-marrow). Recent studies by Choi *et al* (2025, 2026) have generated novel, age-dependent tetrahedral mesh-type skeletal models. Intraskeletal SAFs and photon fluence-to-dose response functions derived from these models have not yet been published, and so the Publication 133 and 155 values remain the most complete set currently available for the present purpose.

### 2.4. Monte Carlo simulation parameters

To generate the electron SAF datasets, the Particle and Heavy Ion Transport code System (PHITS) version 3.35 (Sato *et al* 2024), a benchmarked (Iwamoto *et al* 2022) general-purpose Monte Carlo particle transport simulation code was used with the EGS5 cross section library for electron transport (Hirayama *et al* 2016), where electron cutoff energies were set to 1 keV for electron sources below 1 MeV and 10 keV for electron source energies at or above 1 MeV. Electron source particles were run on a logarithmic scale from 10 keV to 10 MeV, consistent with previous studies (ICRP 2016a, 2023, Schwarz *et al* 2021a, 2021b, Smither *et al* 2026b). Electron collisional absorbed doses were computed in units of Gy per source using the T-Deposit tally in PHITS. To derive the collisional component of the electron SAFs, the PHITS dose tallies were converted from joules to MeV and normalized to the initial particle energy following equation (3).

Bremsstrahlung spectra were computed in units of number of photons per source electron per bin using the T-Product tally in PHITS with bin widths of 10 keV for each simulated source region. The 10 keV bin width was selected to balance spectral resolution against per-bin counting statistics.

Radiation transport simulations were performed on the University of Florida’s HiPerGator supercomputer using 24 parallel cores per simulation from the system’s 19 200 AMD EPYC 9655P Turin 2.5 GHz (4.5 GHz Boost) processors. A systematic approach was adopted to reduce computation time while preserving low statistical uncertainties in the tallied quantities. Particle histories were assigned according to a tiered energy scheme: 10^9^ histories were used for electron sources at or below 80 keV; 5 × 10^8^ histories for sources between 100 keV and 800 keV; and 10^8^ histories for sources between 1 MeV and 10 MeV. Bremsstrahlung photons were counted upon creation but were not transported and did not contribute to the absorbed dose in the target regions. No variance reduction techniques or tally filtering and denoising methods were applied.

### 2.5. Data processing and smoothing

PHITS tally outputs were processed using MATLAB^6^6MATLAB, version R2025b, The MathWorks Inc., Natick, MA, USA.. Collisional and radiative electron SAF components were subsequently derived from the PHITS T-Deposit and T-Product tally results using equations (3) and (7), respectively, and summed to yield the total electron SAF via equation (4).

Our previous study on photon SAFs utilized a weighted Gaussian Process Regression function to smooth the photon SAFs in log-log space. No additional smoothing was applied to the electron SAFs in the present study for two reasons: the photon SAFs used in the computation of radiative electron SAFs were already smoothed as part of our prior work, and source-target combinations yielding nonzero collisional electron SAFs generally exhibited low Monte Carlo relative errors, owing to the proximity of the source and target regions and the energy of the source electron.

### 2.6. Limiting values and low-energy extrapolation

Total electron SAF values were extrapolated to a limiting value of zero energy following the analytical expressions for low-energy SAF behavior established in ICRP Publications 133 and 155 (ICRP 2016a, 2023). The general limiting behavior depends on the geometric relationship between the source and target regions. For non-overlapping source-target pairs, the SAF approaches zero as particle energy tends to zero. For self-absorption cases, where the source and target are the same region, all emitted energy is locally absorbed and the SAF converges to the reciprocal of the target mass:

\begin{equation*}\mathop {\lim }\limits_{E \to 0} {{\Phi }}\left( {{r_{\mathrm{T}}} \leftarrow {r_{\mathrm{S}}},E} \right) = \left\{ {\begin{array}{*{20}{l}} {\frac{1}{{{m_{\mathrm{T}}}}},{\mathrm{if}}\,\,{r_{\mathrm{S}}} = {r_{\mathrm{T}}}} \\ {0,{\mathrm{if}}\,\,{r_{\mathrm{S}}} \ne {r_{\mathrm{T}}}} \end{array}} \right.\end{equation*}

where ${m_{\mathrm{T}}}$ is the mass of the target tissue in kilograms.

For blood as a source region, a fraction of the total blood volume resides within each organ, resulting in partial spatial overlap with target tissues throughout the body. The zero-energy limiting SAF in this case is:

\begin{equation*}\mathop {\lim }\limits_{E \to 0} {{\Phi }}\left( {{r_{\mathrm{T}}} \leftarrow {\mathrm{Blood}},E} \right) = \frac{{{f_{{r_{\mathrm{T}}}}}}}{{{m_{\mathrm{T}}}}}\end{equation*}

where ${f_{{r_{\mathrm{T}}}}}$ is the fraction of total blood volume contained within target tissue ${r_{\mathrm{T}}}$.

An analogous partial overlap condition arises for certain source-target combinations in the alimentary tract, where a defined portion of the source region volume coincides with the target volume. For these geometries, the limiting SAF is determined by the fractional volumetric overlap of the source within the target:

\begin{equation*}\mathop {\lim }\limits_{E \to 0} {{\Phi }}\left( {{r_{\mathrm{T}}} \leftarrow {r_{\mathrm{S}}},E} \right) = \frac{{\frac{{{V_{{\mathrm{S}}{{\mathop \cap \nolimits}}{\mathrm{T}}}}}}{{{V_{\mathrm{S}}}}}}}{{{m_{\mathrm{T}}}}}\end{equation*}

where ${V_{{\mathrm{S}}{{\mathop \cap \nolimits}}{\mathrm{T}}}}$ is the volume of the source region overlapping the target region and ${V_{\mathrm{S}}}$ is the total source region volume. When the source and target tissues share the same density, the volume ratio in equation (10) is equivalently expressed as a ratio of masses.

SAFs at 1 and 5 keV were computed using PCHIP interpolation in log-log space, based on the computed total electron SAFs from 10 keV to 10 MeV and the limiting value at 0 MeV. Pairs for which the calculated SAF at 10 keV was zero were excluded from this procedure: a zero at 10 keV implies zero at all lower energies, and the SAFs at 1 and 5 keV were therefore set to zero directly. For the remaining non-overlapping pairs, the limiting SAF approaches zero as the electron energy approaches zero, but as the logarithm of zero is undefined, a finite lower boundary point is required for the interpolation. A value of 10^−12^ kg^−1^ at 1 eV was adopted for numerical convenience, consistent with the convention used by Schwarz *et al* (2021a, 2021b), the ICRP (2016a, 2023), and our recent photon study (Smither *et al* 2026b).

### 2.7. Quality assurance

To confirm that the total electron SAF values reported in this work are not artifacts of the radiation transport code used, a subset of source-target combinations in the adult male MRCP was independently simulated using Geant4 version 11.01.p02 (Allison *et al* 2016). The phantom was implemented using the G4Tet and G4VPVParameterisation classes. Photons and electrons were transported using G4EMLivermorePhysics with a 1 *μ*m secondary-particle range cutoff, and energy deposition was scored using the G4PSEnergyDeposit class. A total of 10^9^ histories were simulated, and the results were compared against the corresponding PHITS results. In Geant4, the total electron SAFs were computed directly from a single simulation, whereas the PHITS results in this work were obtained by combining separately computed collisional and radiative components. To enable a like-for-like comparison, additional PHITS simulations were performed in which the total electron SAFs were computed directly using the same single-simulation method as in Geant4. Comparing the Geant4 results against both sets of PHITS results (component-based and single-simulation) verifies two things: the agreement between the transport algorithms of the two codes, and the consistency of the component-based method with the single-simulation method for deriving total SAFs.

## 3. Results and discussion

### 3.1. Electron SAFs

In the present study, we have generated comprehensive datasets of electron SAFs (kg^−1^) for the full family of ICRP MRCPs using the PHITS Monte Carlo code. We provide 5332 source-target combinations across 28 energy points (0–10 MeV). Total electron SAFs were computed using the component-based method discussed in equations (3)–(7). Figure 2 illustrates the results from the T-Product tally in PHITS which was used to compute the bremsstrahlung photon yield in various source tissues in the 5-year-old female MRCP. The contributions from the radiative and collisional components to total electron SAFs are shown in figure 3 for selected source-target combinations in the 5-year-old female MRCP. Figure 3 demonstrates that if the source-to-target distance is within the electron range, the electron begins to deposit much of its energy in the target region via collisional interactions, dominating over the radiative component.

**Figure 2.**
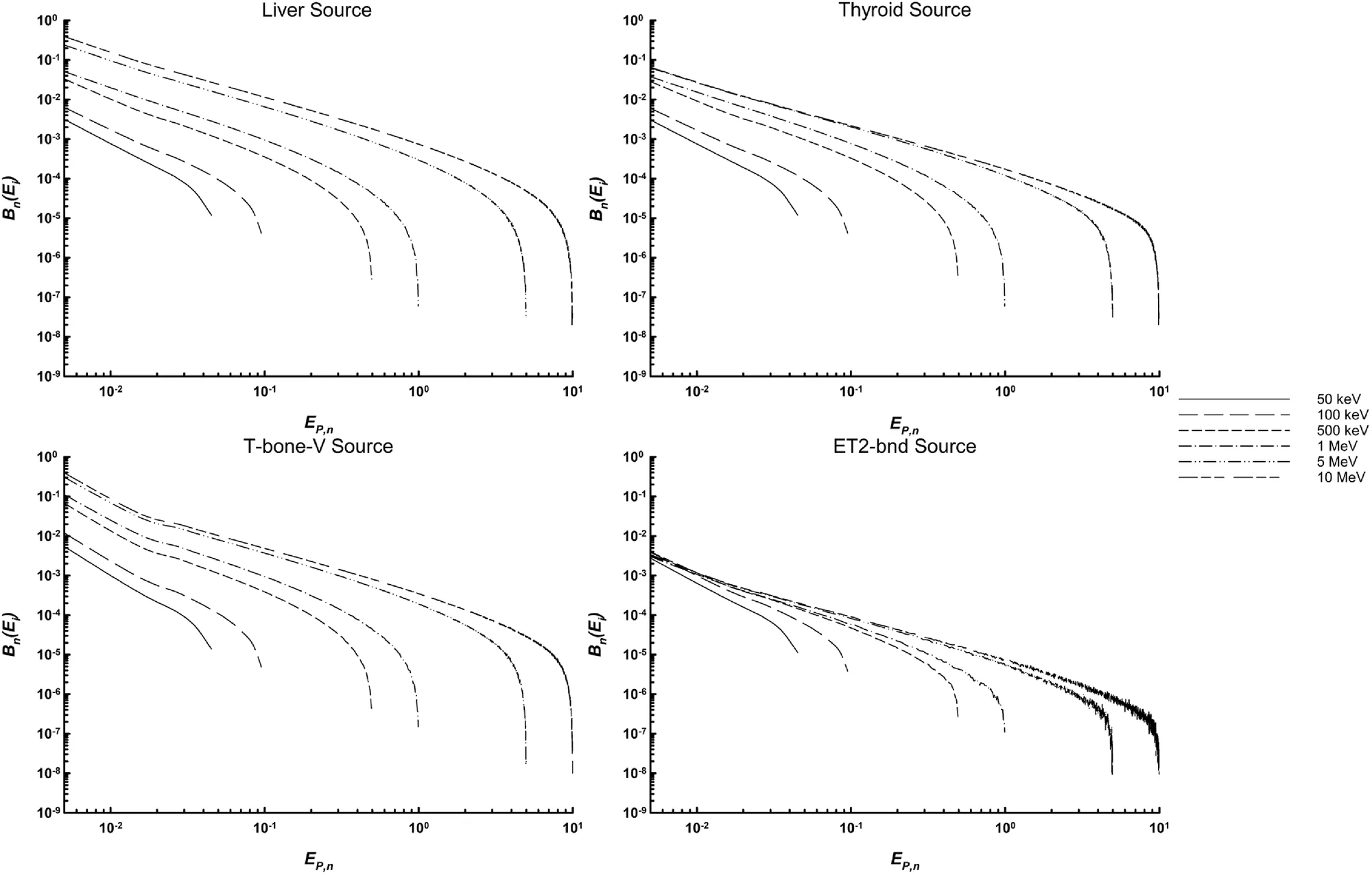
Number of bremsstrahlung photons produced in the nth photon energy bin per source electron of energy ${E_i}$ $({B_n}({E_i}))$ in the 5-year-old mesh-type reference computational phantom (MRCP) for liver, thyroid, trabecular bone volume (T-bone-V), and bound region of the posterior nasal passage (ET2-bnd) source regions for various source electron energies.

**Figure 3.**
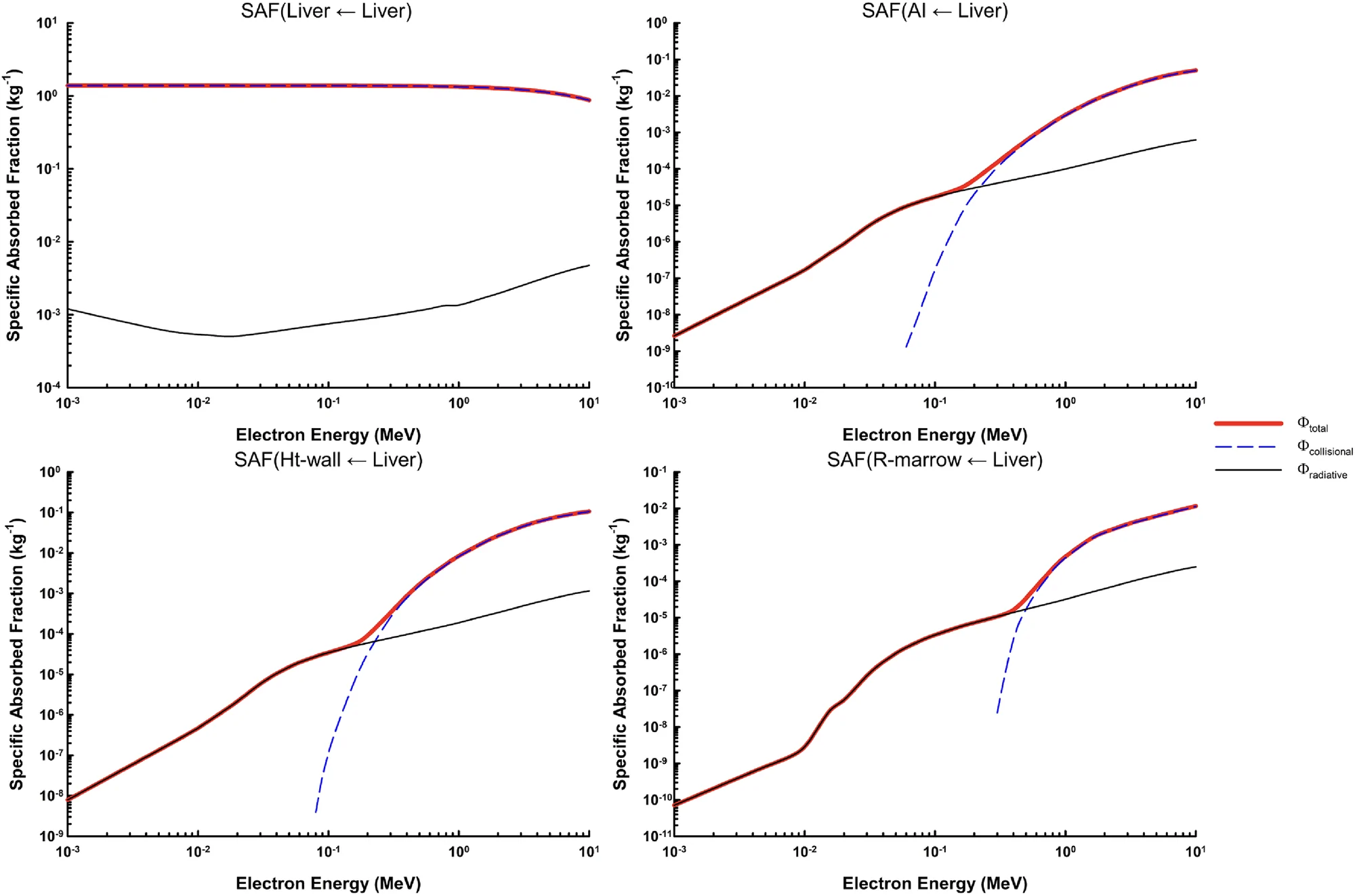
Radiative, collisional, and total electron specific absorbed fractions (SAFs) in the 5-year-old female mesh-type reference computational phantom (MRCP) across various source-target combinations.

Monte Carlo statistical uncertainties were evaluated for the collisional component of every source-target combination by propagating the per-target-region relative errors reported by PHITS. Across all twelve phantoms, 73.8% of non-zero collisional SAFs had a PHITS-reported relative error below 1%, 87.0% below 5%, 90.6% below 10%, 94.3% below 25%, and 96.8% below 50%.

### 3.2. Quality assurance

Quality assurance calculations were performed in the adult male MRCP for blood, thyroid, liver, and brain source regions, with red bone marrow, skin, heart wall, and alveolar interstitium as targets. Figure 4 presents boxplots of the pairwise ratios among three Monte Carlo methodologies: PHITS using a single-simulation method, in which bremsstrahlung photons were generated and tracked within the primary simulation; PHITS using the component-based method employed throughout this study, in which the radiative and collisional components of the electron SAF were computed separately; and Geant4 using the single-simulation method. Each box contains 16 datapoints, excluding pairs in which either value was zero. The three methods were in broad agreement across the full energy range and all source-target combinations. The two PHITS methods gave a mean ratio of 1.10 and a median of 1.00; the two single-simulation implementations tracked one another closely, with a mean of 0.95 and a median of 0.98; and the PHITS component-based method reproduced the Geant4 single-simulation results with a mean of 1.06 and a median of 1.00. Differences from unity likely arise from the high Monte Carlo relative errors that affect distant, low-yield source regions in the single-simulation results, where few bremsstrahlung photons are produced and even fewer deposit their energy in a target region. In the component-based method, the photon SAFs used to compute the bremsstrahlung contribution have lower tally uncertainties and have been smoothed.

**Figure 4.**
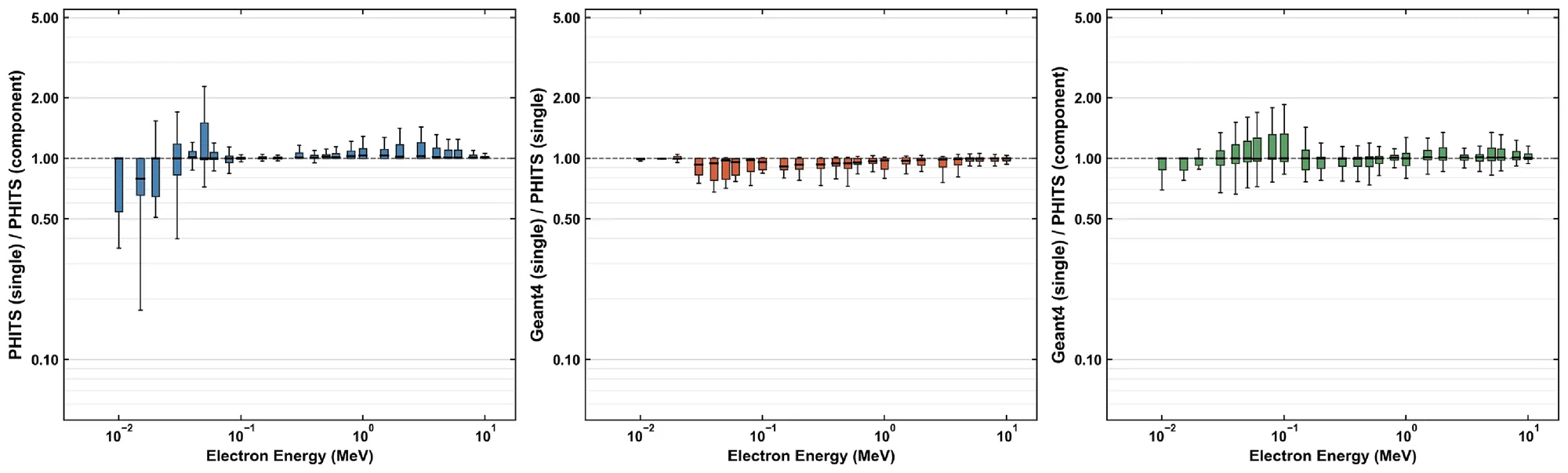
Boxplots of the distributions of ratios of specific absorbed fraction (SAF) values versus electron energy in the adult male mesh-type reference computational phantom (MRCP) for PHITS single-simulation method to PHITS component-based method, Geant4 single-simulation method to PHITS single-simulation method, and Geant4 single-simulation method to PHITS component-based method.

Although the quality assurance comparison presented here was performed using only the adult male MRCP, a recent intercomparison of dosimetric quantities in the ICRP MRCPs using PHITS, Geant4, MCNP6.3, and EGSnrc evaluated both the adult and pediatric phantoms (Shin *et al* 2026). This study reported levels of agreement between PHITS and Geant4 consistent with those obtained here, including for the pediatric MRCPs.

### 3.3. Age-dependent trends in SAFs

Figures 5 and 6 present total electron SAFs for a large solid organ (liver) and a small solid organ (thyroid) to near and distant target regions across the full MRCP age series to assess trends between varying organ sizes. Results for the newborn through 10-year-old pediatric phantoms are shown of the females only for illustrative purposes. Age-dependent trends are generally evident across figures 5 and 6 with SAF values decreasing with increasing age. As the phantom age decreases, inter-organ distances generally become smaller, resulting in higher AFs. In addition, the smaller organ masses in younger phantoms further contribute to higher SAFs. Figures 5 and 6 also demonstrate that some source-target combinations receive dose from both collisional and radiative contributions while some only receive radiative contributions. Table 3 presents median, 5th, and 95th percentiles for pediatric-to-adult ratios of SAFs for self-absorption and crossfire cases. The age dependent trends are evident in table 3 as increasing age brings the median SAF ratio closer to unity.

**Figure 5.**
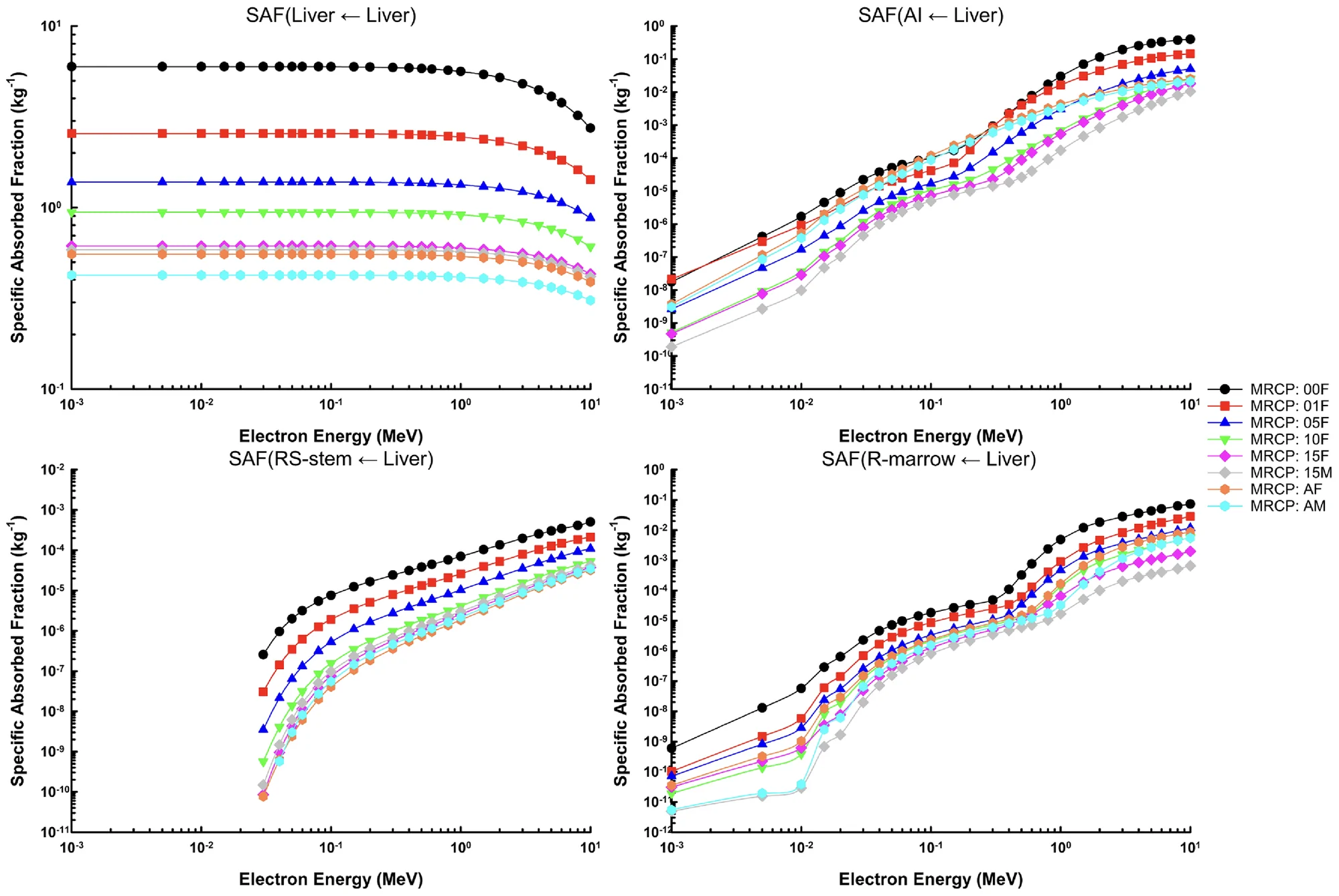
Specific absorbed fractions (SAFs) for electron exposures with the liver as the source and the liver, alveolar interstitium (AI), stem cell layer of the rectosigmoid (RS-stem), and red bone marrow (R-marrow) as target tissues, calculated using the female newborn, 1-, 5-, and 10 year-old, as well as male and female 15 year-old and adult mesh-type reference computational phantoms (MRCPs).

**Figure 6.**
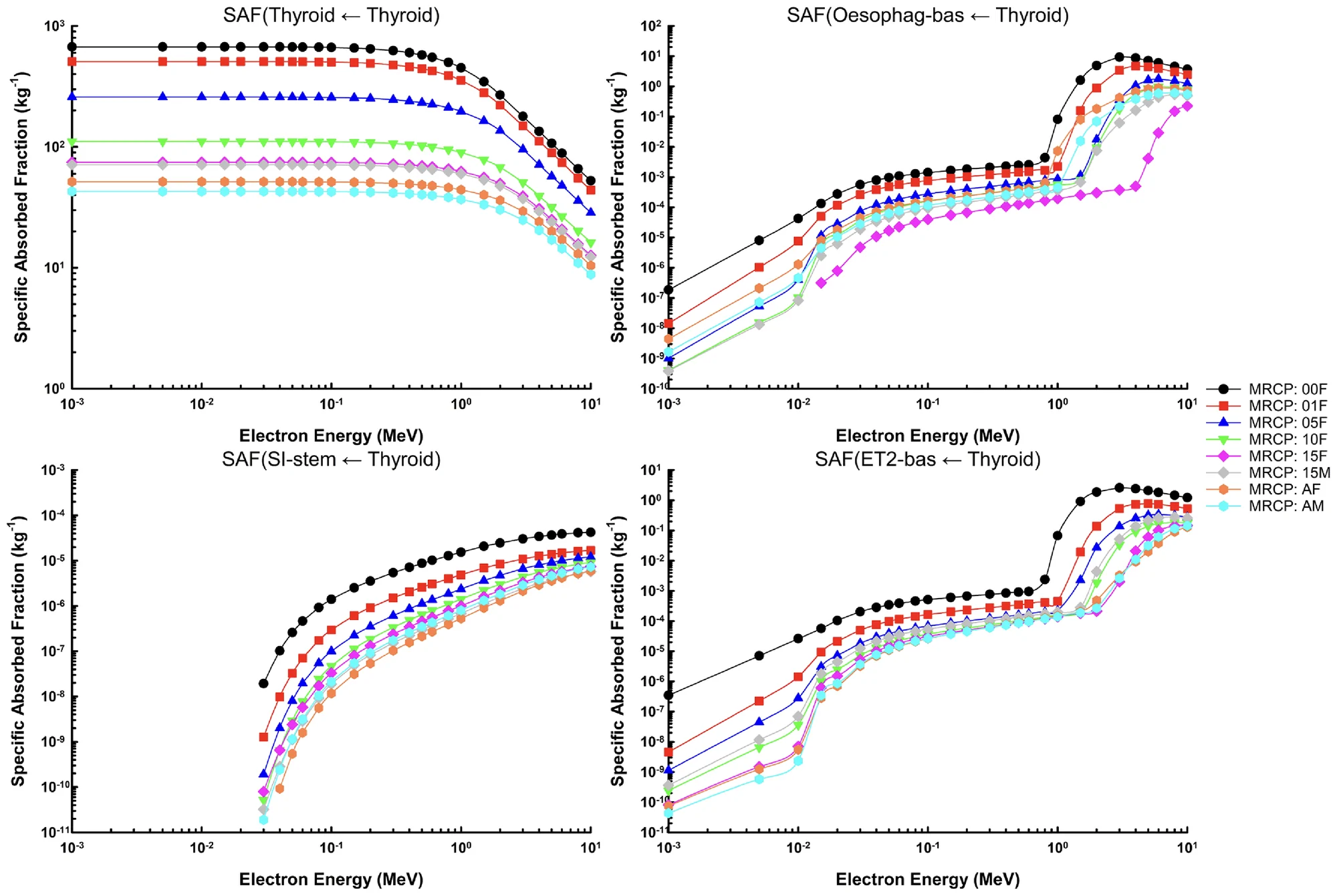
Specific absorbed fractions (SAFs) for electron exposures with the thyroid as the source and the thyroid, basal layer of the oesophagus (Oesophag-bas), stem cell layer of the small intestine (SI-stem), and the basal cells of the posterior nasal passage (ET2-bas) as target tissues, calculated using the female newborn, 1-, 5-, and 10-year-old, as well as male and female 15-year-old and adult mesh-type reference computational phantoms (MRCPs).

**Table 3.** Median, 5th, and 95th percentile for pediatric-to-adult electron specific absorbed fraction (SAF) ratios from self-absorption and crossfire cases. *N* is the number of data points used in the calculations. Values of zero in either the adult or pediatric set were excluded from the calculations.

Pair type	Phantom	*N*	Median	5th percentile	95th percentile
Self-absorption	00F	4620	13.19	3.64	45.77
	00M	4620	15.62	3.57	43.45
	01F	4648	5.20	2.27	14.24
	01M	4648	6.03	2.37	16.31
	05F	4648	2.69	1.45	5.67
	05M	4648	3.10	1.70	7.31
	10F	4648	1.65	0.99	2.83
	10M	4648	1.87	1.27	3.81
	15F	4648	1.13	0.74	1.91
	15M	4648	1.23	0.90	1.95

Crossfire	00F	112 414	20.86	1.77	1657.82
	00M	111 421	25.46	2.73	1945.31
	01F	115 085	7.70	0.87	234.44
	01M	114 064	9.21	1.21	338.38
	05F	114 768	3.36	0.34	45.41
	05M	113 770	3.93	0.51	63.45
	10F	114 052	1.80	0.12	13.36
	10M	113 227	2.08	0.24	18.08
	15F	113 713	1.13	0.08	6.53
	15M	113 136	1.14	0.12	9.44

### 3.4. Comparison of SAFs with ICRP Publications 133 and 155

Ratios of electron SAF values were computed between this work and those reported by the ICRP (2016a, 2023) to investigate differences in various source-target pairs. Values were excluded from the graphs if either the present study or the ICRP produced a value of zero.

Figure 7 investigates changes to electron SAFs after the explicit inclusion of intra-organ blood content in the MRCPs. Publications 133 and 155 SAF employed an SAF scaling procedure in self-absorption geometries to account for missing blood mass in the ICRP voxel phantoms. Publication 155 provides an overview of electron SAF scaling studies performed by Fisher and Snyder (1966), Snyder (1969), Adams (1981), Petoussi-Henss *et al* (2007), and Wayson and Bolch (2018), where a simple linear mass scaling was implemented for charged particles. Some self-absorption geometries show close agreement between this dataset and that reported by the ICRP, which validates the scaling approach employed by the ICRP to account for intra-organ blood content for electron sources.

**Figure 7.**
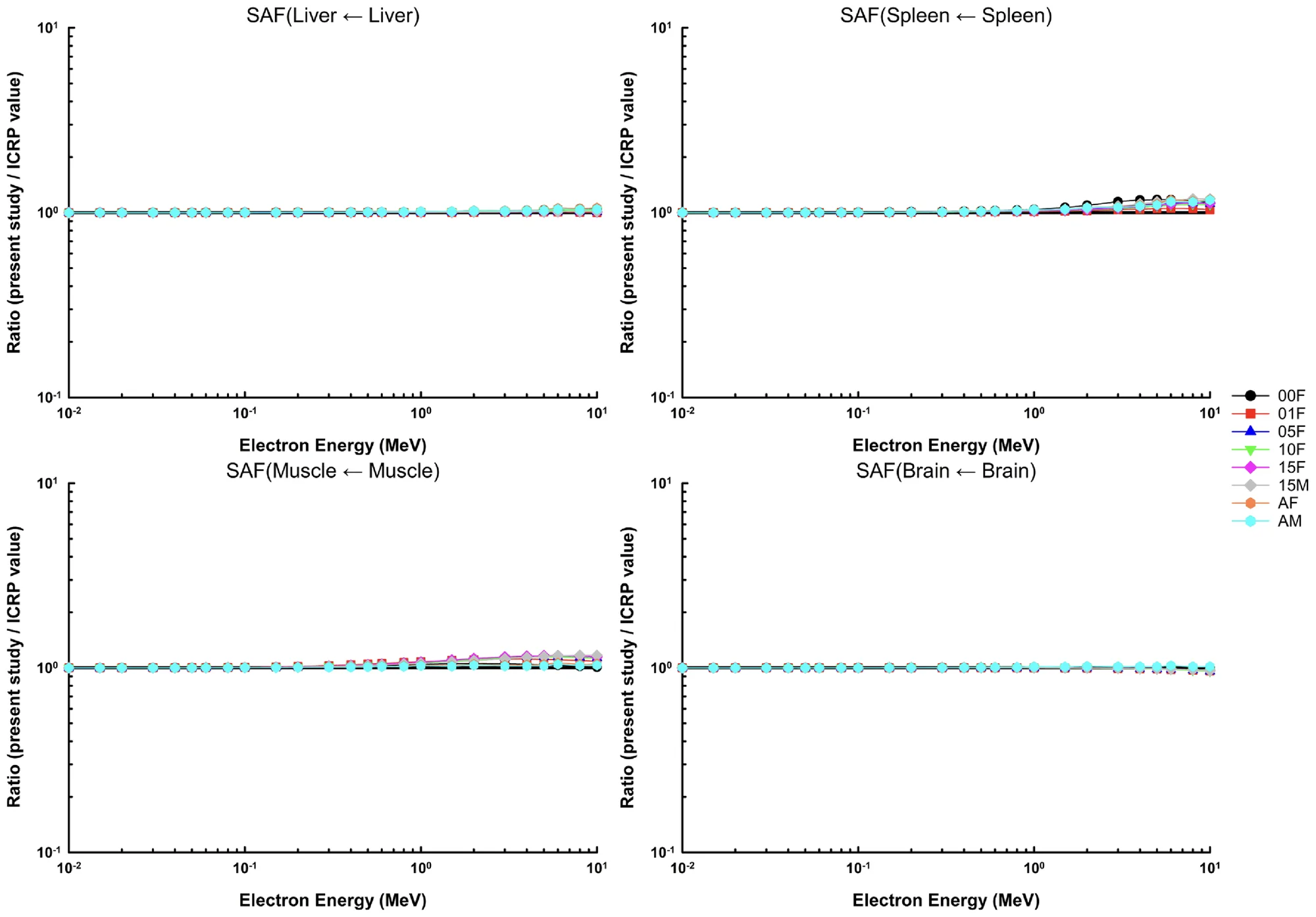
Ratios of specific absorbed fractions (SAFs) of mesh-type computational phantoms (MRCPs) to those from ICRP Publications 133 and 155, shown for source-target pairs involving organs with high blood volume fractions (liver and spleen) and low blood volume fractions (muscle and brain).

Figure 8 presents comparative ratios for muscle sources to bone targets, investigating target regions containing stairstep artifacts in the VRCPs. The trends observed for the red bone marrow and endosteal surface targets are expected, as this study reports lower electron SAFs at lower energies, likely because there are no exposed regions of spongiosa or medullary cavity that an electron born in muscle could reach directly without first passing through cortical bone. In the VRCPs, however, there are instances where the spongiosa and medullary cavity are not fully covered by cortical bone due to stairstep artifacts commonly associated with voxel-based geometries.

**Figure 8.**
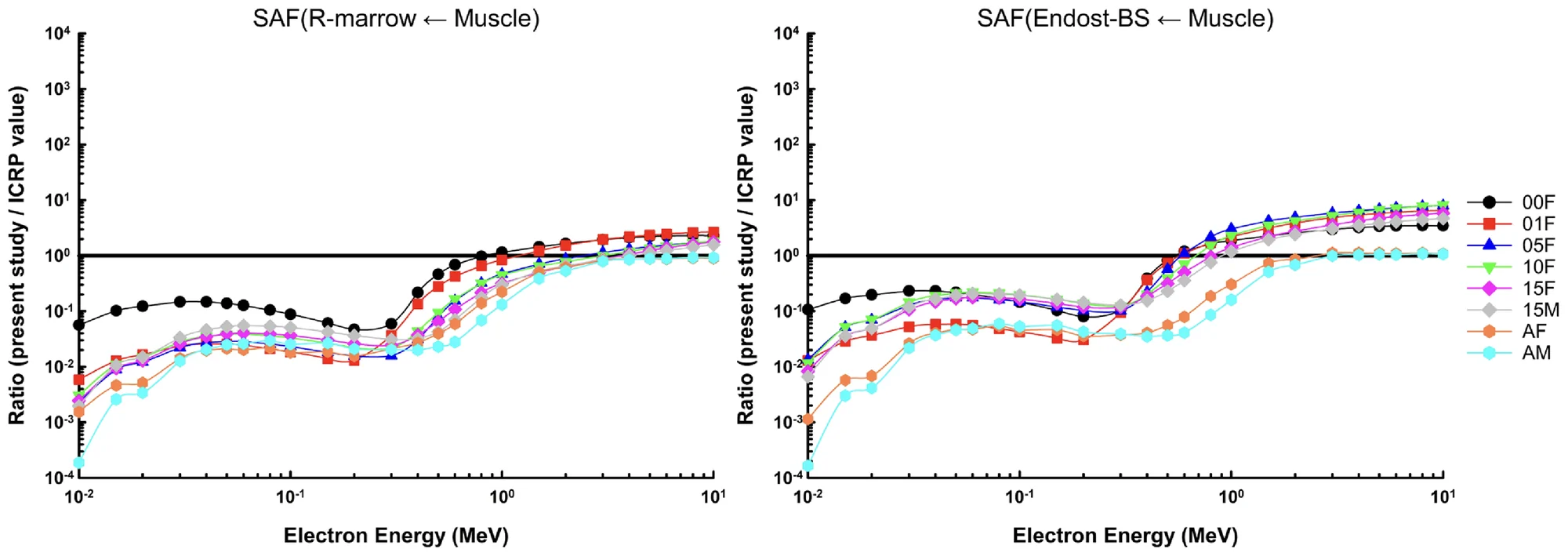
Ratios of specific absorbed fractions (SAFs) of mesh-type computational phantoms (MRCPs) to those from ICRP Publication 133 and 155, shown for muscle sources and its influence on bone targets to investigate the effects of incomplete coverage of the spongiosa and medullary cavity regions in the voxel-type reference computational phantoms (VRCPs) due to stairstep artifacts.

Figure 9 shows the effects of the explicit modeling of micrometer-scale target tissue layers within the alimentary tract within the MRCPs, which are not present within the VRCPs. Certain crossfire cases exhibit pronounced ratio differences at intermediate energies. These arise from shifts in the energy at which the collisional SAF component begins to dominate over the radiative component where the transition occurs at a lower energy in the present study compared to the ICRP data, though the overall shape of the energy dependence remains consistent between the two datasets. These differences are primarily attributable to updated alimentary tract geometries in the MRCPs, and analogous behavior is observed in other source-target combinations where the explicit inclusion of intra-organ blood in the MRCPs increases organ volumes, effectively reducing source-to-target separation.

**Figure 9.**
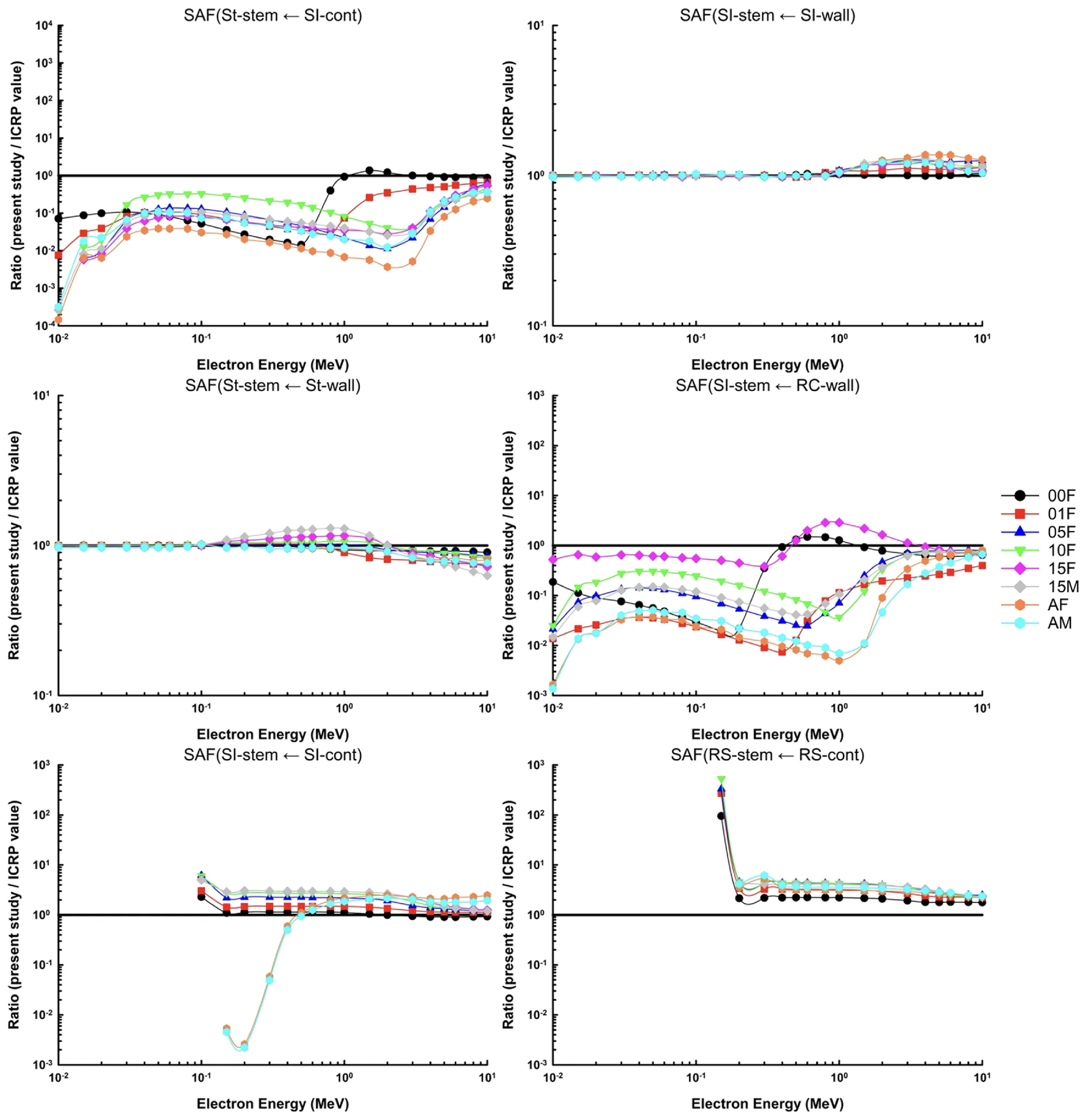
Ratios of specific absorbed fractions (SAFs) of mesh-type computational phantoms (MRCPs) to those from ICRP Publication 133 and 155, shown for source-target pairs involving the micrometer-scale target layers of the alimentary tract as explicitly defined in the MRCPs.

Figure 10 provides ratios for organs that underwent substantial anatomical revisions to their geometries during MRCP development. Organ-dependent differences are observed. For the thyroid, the electron SAFs presented in this study agree with the ICRP values across the energy range. Revisions to the geometries of the oral cavity, oral mucosa, and the lens of the eye yield distinct differences from the ICRP values.

**Figure 10.**
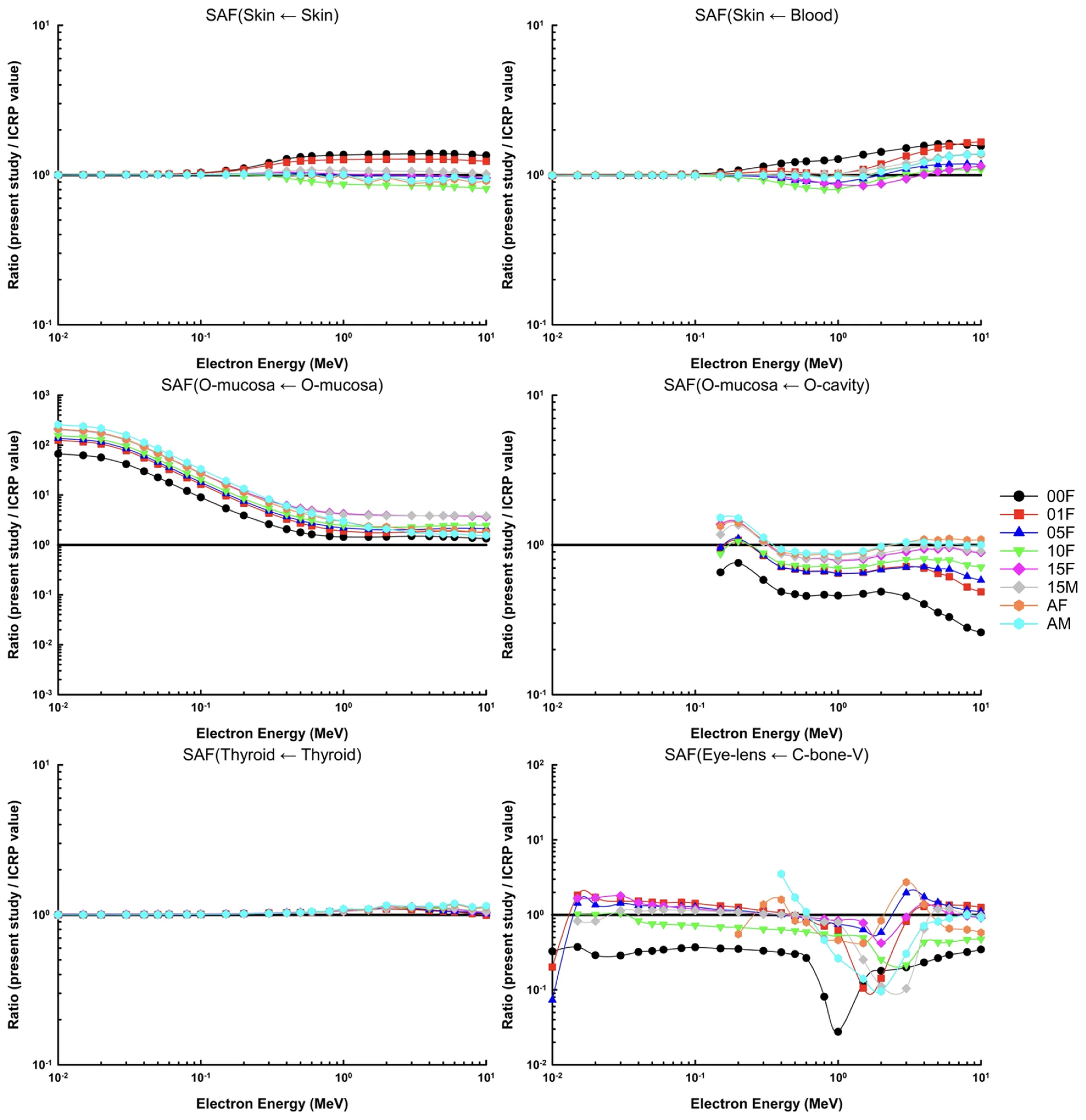
Ratios of specific absorbed fractions (SAFs) of mesh-type computational phantoms (MRCPs) to those from ICRP Publications 133 and 155, shown for source-target pairs involving organs and tissues whose geometry was modified during the development of the MRCPs from the voxel-type reference computational phantoms (VRCPs).

For many crossfire cases, low-energy differences largely reflect divergent low-energy extrapolation strategies. The ICRP adopted the method of Schwarz *et al* (2021a, 2021b), which replaced some Monte Carlo results with enforced linear behavior in this energy region regardless of statistical quality as well as forcing non-zero values up to the limiting value for crossfire cases. The present study retains calculated SAF values at the low-energy points and if a zero-valued SAF were found at a low energy, the zero was maintained.

For self-absorption geometries across 92 matched source-target pairs (figure 11), SAFs computed from the 5-year-old female MRCP show broad agreement with the linearly scaled values from ICRP Publication 155 across the entire electron energy range, with median ratios close to unity and narrow interquartile ranges at low source particle energies. Larger interquartile ranges at higher energies are driven by differences in SAFs for blood sources and overlapping source-target combinations in the alimentary and respiratory tracts at these higher energies. For crossfire geometries across 3142 matched source-target pairs, the median ratio lies below unity across the energy range, with considerable spread that increases at lower energies. The observed disagreement likely reflects a combination of factors: differences in organ volumes and inter-organ distances between the two phantoms, differences in bremsstrahlung photon yield, differences in lower-energy extrapolation methods, and differences in the photon datasets used to compute the radiative electron SAF.

**Figure 11.**
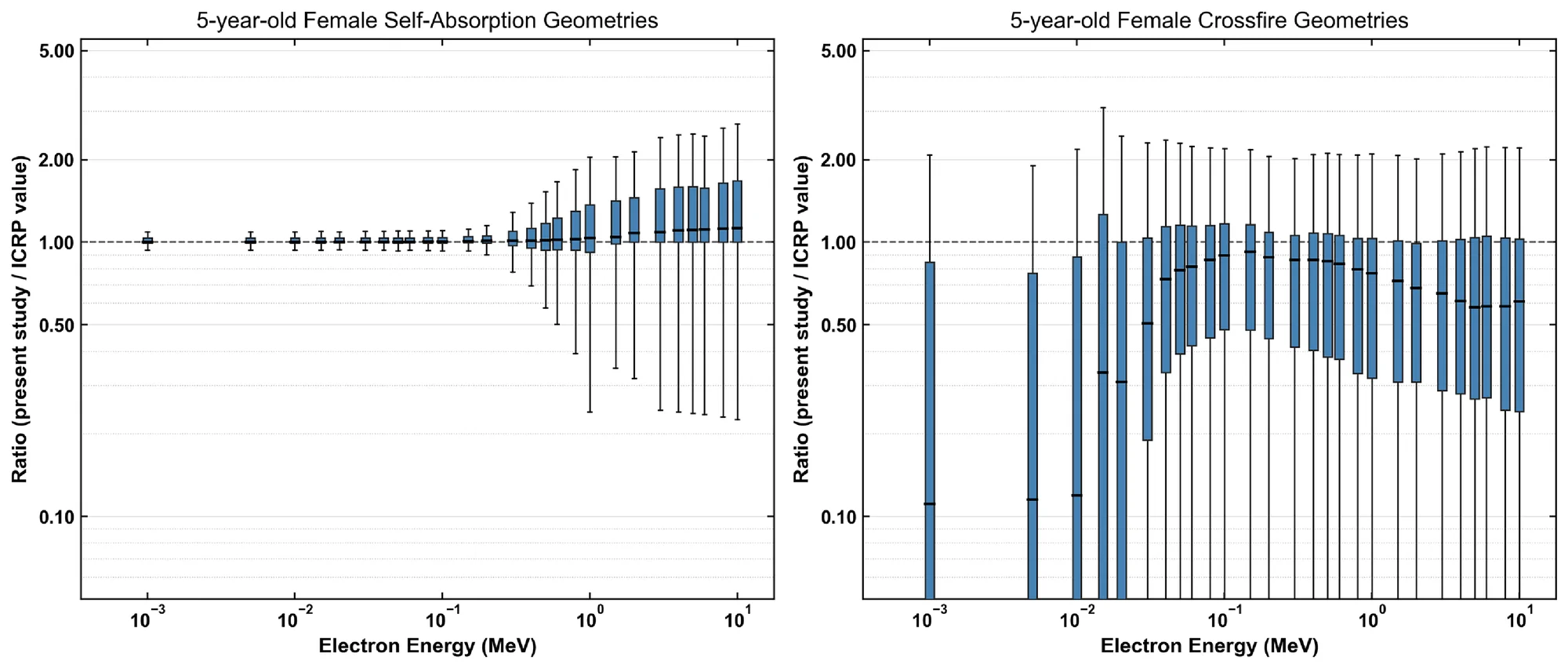
Boxplots of the distributions of ratios of specific absorbed fraction (SAF) values versus electron energy in the 5-year-old female for self-absorption geometries and crossfire geometries. Boxes are comprised of 92 matched source-target pairs for self-absorption geometries, and 3142 matched source-target pairs for crossfire geometries.

*S* values were calculated for Y-90, I-131, and Lu-177 for a liver source using the 5 year-old female MRCP and VRCP following equation (2) and emission spectra taken from ICRP Publication 107 (ICRP 2008). Table 4 presents these for liver, kidneys, skin, and stem cells of the small intestines as targets. There is relatively good agreement between the MRCP and VRCP values for both self-absorption and crossfire cases. The largest difference between the two phantoms was found for a Y-90 liver source to the stem cell layer of the small intestines. For I-131 and Lu-177 for this crossfire case, the photon contribution to the total *S* value is much greater than the electron component while for Y-90, the electron contribution to the *S* value is greater than the photon component. Additionally, Y-90 has a higher average beta-particle energy compared to I-131 and Lu-177.

**Table 4.** Radionuclide *S* values calculated for selected radionuclides and source-target combinations for the 5-year-old female mesh-type reference computational phantom (MRCP) and the 5-year-old female voxel-type reference computational phantom (VRCP).

	*S* values (mGy/MBq-s)							
	*S* (Liver ← Liver)		*S* (Kidneys ← Liver)		*S* (Skin ← Liver)		*S* (SI-stem ← Liver)	
Radionuclide	MRCP	VRCP	MRCP	VRCP	MRCP	VRCP	MRCP	VRCP
Y-90	1.98 × 10^−04^	1.96 × 10^−04^	2.78 × 10^−07^	3.52 × 10^−08^	3.47 × 10^−09^	3.59 × 10^−09^	1.99 × 10^−08^	5.60 × 10^−08^
I-131	5.33 × 10^−05^	5.21 × 10^−05^	2.51 × 10^−06^	2.52 × 10^−06^	3.55 × 10^−07^	3.58 × 10^−07^	1.02 × 10^−06^	1.08 × 10^−06^
Lu-177	3.38 × 10^−05^	3.36 × 10^−05^	2.53 × 10^−07^	2.58 × 10^−07^	3.09 × 10^−08^	3.17 × 10^−08^	1.01 × 10^−07^	1.09 × 10^−07^

A limitation of this study is that it did not assess comprehensive differences in radionuclide *S* values between the MRCPs and the VRCPs as the focus was on the calculation of electron SAFs only. Comprehensive *S* values will be computed in the future using the photon and electron SAFs calculated from the MRCPs.

## 4. Conclusion

In this study, comprehensive datasets of electron SAFs have been generated for the full family of ICRP MRCPs using the PHITS Monte Carlo code, providing 5332 source-target combinations across 28 energy points spanning 0–10 MeV. Total electron SAFs were computed by separating collisional and radiative contributions, and results for the adult male MRCP were independently verified against Geant4 calculations, with broad agreement observed across the full energy range for all tissues investigated. The component-based method for computing electron SAFs demonstrated a clear advantage over brute-force approaches at low electron energies for crossfire cases, where insufficient bremsstrahlung statistics in direct simulations would otherwise yield zero-valued SAFs. Age-dependent trends are generally observed across the MRCP family, with SAF values increasing with decreasing age, reflecting the smaller organ masses and reduced inter-organ distances in the younger phantoms. Comparisons with values reported in ICRP Publications 133 and 155 reveal agreement for the important self-absorption geometries with discrepancies in crossfire cases due to differences in phantom anatomy, bremsstrahlung photon production results, low-energy extrapolation methodology, and the underlying photon SAF datasets used to compute the radiative component. The electron SAF datasets produced in this work provides a foundation for internal dosimetry calculations across the full ICRP MRCP family and is intended to support the development of updated dose coefficients.

## Data Availability

The data that support the findings of this study are openly available at the following URL/DOI: https://github.com/wsmither17/PMB-MRCP_electron_SAFs.git (Smither *et al* 2026a).
